# Exposure to chemical components of fine particulate matter and ozone, and placenta-mediated pregnancy complications in Tokyo: a register-based study

**DOI:** 10.1038/s41370-021-00299-4

**Published:** 2021-02-18

**Authors:** Takehiro Michikawa, Seiichi Morokuma, Shin Yamazaki, Akinori Takami, Seiji Sugata, Ayako Yoshino, Yuki Takeda, Kazushige Nakahara, Shinji Saito, Junya Hoshi, Kiyoko Kato, Hiroshi Nitta, Yuji Nishiwaki

**Affiliations:** 1 Department of Environmental and Occupational Health, School of Medicine, Toho University, Ota-ku, Tokyo Japan; 2 Department of Health Sciences, Graduate School of Medical Sciences, Kyushu University, Higashi-ku, Fukuoka Japan; 3 Centre for Health and Environmental Risk Research, National Institute for Environmental Studies, Tsukuba, Ibaraki Japan; 4 Centre for Regional Environmental Research, National Institute for Environmental Studies, Tsukuba, Ibaraki Japan; 5 Department of Obstetrics and Gynaecology, Graduate School of Medical Sciences, Kyushu University, Higashi-ku, Fukuoka Japan; 6 Tokyo Metropolitan Research Institute for Environmental Protection, Koto-ku, Tokyo Japan

**Keywords:** Particulate matter, Chemical component, Placenta-mediated pregnancy complications, Placentation

## Abstract

**Background:**

Maternal exposure to fine particulate matter (PM_2.5_) was associated with pregnancy complications. However, we still lack comprehensive evidence regarding which specific chemical components of PM_2.5_ are more harmful for maternal and foetal health.

**Objective:**

We focused on exposure over the first trimester (0–13 weeks of gestation), which includes the early placentation period, and investigated whether PM_2.5_ and its components were associated with placenta-mediated pregnancy complications (combined outcome of small for gestational age, preeclampsia, placental abruption, and stillbirth).

**Methods:**

From 2013 to 2015, we obtained information, from the Japan Perinatal Registry Network database, on 83,454 women who delivered singleton infants within 23 Tokyo wards (≈627 km^2^). Using daily filter sampling of PM_2.5_ at one monitoring location, we analysed carbon and ion components, and assigned the first trimester average of the respective pollutant concentrations to each woman.

**Results:**

The ORs of placenta-mediated pregnancy complications were 1.14 (95% CI = 1.08–1.22) per 0.51 μg/m^3^ (interquartile range) increase of organic carbon and 1.11 (1.03–1.18) per 0.06 μg/m^3^ increase of sodium. Organic carbon was also associated with four individual complications. There was no association between ozone and outcome.

**Significance:**

There were specific components of PM_2.5_ that have adverse effects on maternal and foetal health.

## Introduction

Exposure to fine particulate matter (PM_2.5_) in the ambient atmosphere has adverse health effects across all generations [1]. Women of reproductive age are not exceptions. There is evidence that exposure to PM_2.5_ during pregnancy is associated with obstetric complications that threaten the health of pregnant mothers, such as hypertensive disorders of pregnancy (including preeclampsia) and placental abruption [2–5]. In addition, maternal exposure to PM_2.5_ appears to have harmful effects on the next generation; for example, PM_2.5_ exposure during pregnancy was associated with foetal growth restriction that resulted in the birth of small for gestational age (SGA) children [6, 7], and was linked to the occurrence of stillbirths [8]. Although the accumulating findings suggest that total PM_2.5_ (general mixture) influences maternal and foetal health, we still lack comprehensive evidence regarding which specific chemical components of PM_2.5_ are more harmful for their health [9]. Thus, understanding the association between individual PM_2.5_ components and perinatal health may provide information of value to policy making for environmental health.

The placenta is a temporal organ, and normal placentation is absolutely essential for foetal development and growth, while also contributing to maternal health [10]. Conversely, abnormal placentation during the first trimester is related to adverse maternal and foetal conditions called placenta-mediated pregnancy complications, including SGA, preeclampsia, placental abruption, and stillbirth [11–13]. As aforementioned, each of these four complications has been reported to be individually associated with maternal exposure to PM_2.5_ [2, 5–8]. Therefore, in the present study, we focused on PM_2.5_ exposure over the first trimester, which is an important period of placentation, and investigated the association with placenta-mediated pregnancy complications, with the aim of elucidating the relationship between PM_2.5_ exposure and placental toxicity, and seeking a pathogenic foundation common to individual complications. In light of the fact that PM_2.5_ may be a common risk factor for four individual complications, few studies have examined the association between PM_2.5_ exposure and placenta-mediated pregnancy complications as a composite outcome. Also, it seems likely that ozone is a risk factor for placenta-mediated pregnancy complications, because the potential maternal and foetal health effects of ozone exposure were indicated [14].

In sum, the aim of this study was to investigate the association between first-trimester exposure to specific PM_2.5_ chemical components and ozone, and placenta-mediated pregnancy complications in Tokyo, one of the world’s major cities.

## Methods

### Study area and participants

Our study area included 23 Tokyo wards located on the east side of Tokyo, with a total land area of roughly 627 square kilometres. The population of the 23 wards was about 9,272,000 as of 1 October 2015 [15]. Tokyo has a humid subtropical climate, and the annual average temperature is around 16 °C. The average annual urban background concentrations of PM_2.5_ in the study area (e.g., 16.9 μg/m^3^ in 2014) is higher than the nationwide background concentrations of PM_2.5_ (e.g., 14.7 μg/m^3^ in 2014) [16].

We collected data on all the live births and stillbirths after 22 weeks of gestation at 39 cooperating hospitals within the study area, from January 2013 to December 2015 (a total of 89,417 births), which were extracted from the Japan Perinatal Registry Network database, a hospital-based registry (mainly university hospitals and local general hospitals) managed by the Japan Society of Obstetrics and Gynaecology. Details of the database are described elsewhere [17]. The database included roughly two-fifths of the total births in the study area for the study period. The data are routinely input by attending physicians via a standardised electronic form, and checked with respect to uniform coding specifications and diagnostic criteria for complications by the Perinatal Committee of the Society [18]. We used anonymised information on maternal age, height, weight, parity, gestational age, smoking habits and alcohol drinking, infertility treatment, medical history, diagnoses of obstetric complications, such as preeclampsia and placental abruption, mode of delivery, neonatal records, and the hospital at which the woman delivered.

From the 89,417 births including multiple births, we firstly restricted to 85,496 singleton births (85,496 women). Then, we excluded 254 women without information on (at least one of) age at delivery, infant sex, and parity, and 132 women who delivered infants born after 42 weeks of gestation. In Japan, some women return to their hometown near term and deliver at a hospital near their parents’ home (known as *‘satogaeri’* in Japanese). Therefore, as such women may not have resided in our target area during early pregnancy, we also excluded them (*n* = 1656) to avoid exposure misclassification during the first trimester. In the end, 83,454 women (22–41 weeks of gestation) were included in the analysis. The study protocol was approved by the Ethics Committee of Faculty of Medicine, Toho University [A18049_A20024].

### Measurement of fine particulate matter and its chemical components

We obtained air pollutant data, including the daily mean concentrations of total PM_2.5_ and the maximum 8-h mean concentrations of ozone, measured at an urban background monitoring station (Harumi monitoring station, 35.4°N, 139.5°E) (Fig. S1) near the monitoring point of PM_2.5_ chemical components, from the Japan National Institute for Environmental Studies’ atmospheric environment database. The β-ray absorption method was used for PM_2.5_ measurement, and the ultraviolet absorption method was used for ozone measurement. Since there are several urban background monitoring stations in the study area, we confirmed that the PM_2.5_ and ozone concentrations at the Harumi station were strongly correlated with those at the other stations (Pearson’s correlation coefficients >0.9), and thus considered that the background concentrations of PM_2.5_ and ozone were spatially homogeneous within our target area. Finally, we obtained the daily mean ambient temperatures from the Japan Meteorological Agency.

From April 2013 to the end of the study period in December 2015, daily sampling of PM_2.5_ (from 10:00 a.m. to 9:00 a.m. of the next day) was performed at the Tokyo Metropolitan Research Institute for Environmental Protection, located in the southeast of the study area (35.7°N, 139.8°E, roughly 5 km east of the Harumi monitoring station) (Fig. S1). This institute is considered able to measure the typical ambient air pollutant concentrations in the study area [19]. Using an FRM-2000 sampler (Rupprecht and Patashnick, Albany, NY, USA), samples were collected on a quartz-fibre filter (47 mm diameter, 2500 QAT-UP; Pall Life Sciences, Port Washington, NY, USA), following the Federal Reference Methods of the US Environmental Protection Agency [9]. Based on the standardised protocol of the Ministry of the Environment, Japan [20], total carbon, including organic carbon (OC) and elemental carbon (EC), was analysed using a dual optical carbon analyser (OCEC Carbon Aerosol Analyzer; Sunset Laboratory Inc., Tigard, OR, USA); and the ions, including nitrate, sulphate, ammonium, chloride, sodium, potassium and calcium, were analysed using an ion chromatograph (Dionex ICS-5000; Thermo Fisher Scientific Inc., Waltham, MA, USA). Details of the air sampling and chemical analysis are described elsewhere [21].

As we did not have information on participants’ residential address, we assigned measurements at the Harumi monitoring station (total PM_2.5_ and ozone) and Tokyo Metropolitan Research Institute (PM_2.5_ components) to all the women. Based on the birth date and gestational age determined by ultrasound findings during early pregnancy, we estimated the period of the first trimester (0–13 weeks of gestation), and calculated the first trimester average of the respective pollutant concentrations as the main exposure. As the control exposure windows, we used the average concentrations over the 3 months before pregnancy and the second trimester average concentrations (14–27 weeks of gestation).

### Placenta-mediated pregnancy complications

We defined placenta-mediated pregnancy complications as a composite outcome, including SGA, preeclampsia, placental abruption and/or stillbirth (antepartum death) [12]. SGA was defined as birth weight below the 10th percentile according to gestational age, infant sex and parity (0 or ≥1), based on the Japanese neonatal anthropometric chart [22]. Preeclampsia and placental abruption were based on the diagnosis by attending physicians. At the study period, preeclampsia was diagnosed in women presenting with a new onset of hypertension (systolic blood pressure ≥140 mmHg and/or diastolic blood pressure ≥90 mmHg) after 20 weeks of gestation and proteinuria (two or more dipstick readings of 3+ or greater, or a 24-h urine collection containing at least 300 mg of protein) [23].

### Statistical methods

All statistical analyses were performed with Stata15 for Windows (Stata Corporation, College Station, TX, USA).

The women were categorised into five groups according to their level of exposure to total PM_2.5_, its chemical components and ozone during the first trimester. The data in this study had a hierarchical structure that the participants were nested within hospitals. To explore the association between exposure to pollutants during the first trimester, and placenta-mediated pregnancy complications, as a composite outcome and in terms of individual complications, we considered this hierarchical structure, and applied a multilevel logistic regression model with the hospital as a random effect. With the lowest concentration group as the reference, first the odds ratios (ORs) and 95% confidence intervals (CIs) of placenta-mediated pregnancy complications were estimated, after adjustment for maternal age at delivery (<25, 25–29, 30–34, ≥35 years), birth year (2013, 2014, 2015) and season of conception (spring, summer, autumn, winter). Then, the smoking habits and alcohol drinking (yes, no, missing), prepregnancy body mass index (<18.5, 18.5–24.9, ≥25 kg/m^2^, missing), current history of diabetes/gestational diabetes (yes, no), infertility treatment (no, ovarian stimulation/artificial insemination by sperm from husband, assisted reproductive technology) and parity (0, 1, ≥2) were included in the model as potential confounding factors. For the association between PM_2.5_ and a composite outcome, we checked whether natural cubic spline model (non-linear model) was superior to a linear model, and observed no significant divergence from linearity. Then, the ORs per interquartile range increase (IQR) in the pollutant concentrations were also estimated. We treated pollutant exposure over the 3 months before pregnancy and over the second trimester as the control exposure windows, and conducted the same analysis.

We investigated the respective associations between first-trimester exposure to specific PM_2.5_ chemical components one-by-one and placenta-mediated pregnancy complications (single-component model). Then, we constructed a multi-component model, which included all components associated with the composite outcome in the single-component models, to assess which components had an independent effect on the outcome. We further checked whether the first trimester was a sensitive period for the outcome occurrence (adjusted for exposure over the 3 months before pregnancy and the second trimester), and whether the observed association was affected by the confounding of total PM_2.5_ and ozone, or temperature (adjusted for average concentrations of PM_2.5_ and ozone, and average temperature during the first trimester). Also, since past history of preeclampsia, placental abruption, stillbirth and/or foetal growth restriction were risk factors for placenta-mediated pregnancy complications [13], sensitivity analyses were performed after excluding women with such past histories, and after restricting to nulliparae. Finally, we used the average concentrations over the 8th–12th gestational weeks (i.e., the early stage of placentation) as a specific exposure window for the pathogenesis of placenta-mediated pregnancy complications [24].

## Results

Mean age at delivery of the 83,454 women was 33.7 years (standard deviation (SD) = 5.0), with the percentage ≥35 years accounting for 45.7%. The distribution of characteristics did not differ substantially among the five groups based on total PM_2.5_ concentrations over the first trimester (Table 1). In the overall population, placenta-mediated pregnancy complications were identified in 8663 women (10.4%), with the numbers for individual complications being 6606 for SGA, 1104 for preeclampsia, 1308 for placental abruption, and 323 for stillbirth.

**Table 1:** Characteristics of 83,454 women according to quintiles of total PM_2.5_ concentrations over the first trimester (0–13 weeks of gestation) in 23 Tokyo wards, from 2013 to 2015.

	Quintile									
	1 (lowest)		2		3		4		5 (highest)	
Variables	*n*	%	*n*	%	*n*	%	*n*	%	*n*	%
Total PM_2.5_, median (interquartile range) (μg/m^3^)	14.0 (13.1–14.3)		15.2 (14.9–15.5)		16.1 (15.8–16.4)		17.8 (17.5–18.5)		21.3 (20.5–21.7)	
No. of women	16,616		16,736		16,678		16,714		16,710	
Maternal age at delivery (years)										
<25	680	4.1	661	4.0	661	4.0	594	3.6	638	3.8
25–29	2707	16.3	2691	16.1	2762	16.6	2723	16.3	2631	15.8
30–34	5723	34.4	5697	34.0	5782	34.7	5699	34.1	5681	34.0
≥35	7506	45.2	7687	45.9	7473	44.8	7698	46.1	7760	46.4
Parity										
0	9918	59.7	10,178	60.8	10,060	60.3	10,020	60.0	10,185	61.0
1	5285	31.8	5158	30.8	5190	31.1	5222	31.2	5064	30.3
≥2	1413	8.5	1400	8.4	1428	8.6	1472	8.8	1461	8.7
Smoking habits										
No	12,757	95.1	13,192	96.9	13,109	97.1	13,443	97.2	13,312	95.7
Yes	651	4.9	416	3.1	390	2.9	393	2.8	592	4.3
Alcohol drinking										
No	11,676	94.4	11,693	96.5	11,642	96.5	11,815	97.1	11,311	95.6
Yes	688	5.6	425	3.5	429	3.6	355	2.9	525	4.4
Prepregnancy body mass index (kg/m^2^)										
<18.5	2768	19.5	2774	19.5	2792	19.7	2678	18.9	2794	19.9
18.5–24.9	10,352	72.9	10,412	73.3	10,341	73.1	10,412	73.7	10,235	72.7
≥25.0	1082	7.6	1015	7.2	1015	7.2	1046	7.4	1049	7.5
Current history of diabetes/gestational diabetes										
No	15,750	94.8	15,924	95.2	15,809	94.8	15,792	94.5	15,850	94.9
Yes	866	5.2	812	4.9	869	5.2	922	5.5	860	5.2
Infertility treatment										
No	13,881	83.5	14,131	84.4	14,179	85.0	13,965	83.6	13,998	83.8
Ovarian stimulation/artificial insemination by sperm from husband	1047	6.3	1028	6.1	952	5.7	1149	6.9	1165	7.0
Assisted reproductive technology	1688	10.2	1577	9.4	1547	9.3	1600	9.6	1547	9.3
Past history of complications										
Foetal growth restriction	54	0.3	62	0.4	79	0.5	80	0.5	72	0.4
Preeclampsia	29	0.2	19	0.1	22	0.1	33	0.2	28	0.2
Placental abruption	38	0.2	36	0.2	37	0.2	41	0.3	42	0.3
Stillbirth	83	0.5	96	0.6	86	0.5	101	0.6	83	0.5
Placenta-mediated pregnancy complications as a composite outcome	1669	10.0	1792	10.7	1761	10.6	1783	10.7	1658	9.9
Small for gestational age	1253	7.6	1368	8.2	1343	8.1	1371	8.2	1271	7.6
Preeclampsia	213	1.3	222	1.3	215	1.3	234	1.4	220	1.3
Placental abruption	263	1.6	286	1.7	272	1.6	253	1.5	234	1.4
Stillbirth	77	0.5	53	0.3	65	0.4	67	0.4	61	0.4

Summary statistics of pollutants exposure over the first trimester are shown in Table 2. The average exposure to total PM_2.5_ was 16.8 (SD = 2.6) μg/m^3^. Exposure to PM_2.5_ chemical components was assigned to 67,706 women, because these measurements only began in April 2013, which the study began in January of that year. The major components were total carbon (OC and EC), nitrate, sulphate and ammonium, and their average exposures were 4.0 (0.5), 1.4 (0.8), 2.8 (1.0), and 1.5 (0.3) μg/m^3^, respectively. The correlation coefficient between OC and EC was 0.76.

**Table 2:** Summary statistics and correlations of pollutant exposures over the first trimester (0–13 weeks of gestation) in 23 Tokyo wards, from 2013 to 2015.

	No. of women	Mean (SD)	Percentile				Pearson’s correlation coefficient												
			25	50	75	IQR	Total PM_2.5_	Total carbon	OC	EC	Nitrate	Sulphate	Ammonium	Chloride	Sodium	Potassium	Calcium	Ozone	Temperature
Total PM_2.5_ (μg/m^3^)	83,454	16.8 (2.6)	14.9	16.1	18.5	3.62	1												
PM_2.5_ components (μg/m^3^)^a^																			
Total carbon	67,706	4.0 (0.5)	3.7	4.1	4.3	0.62	−0.17*	1											
OC	67,706	2.7 (0.4)	2.4	2.7	2.9	0.51	−0.22*	0.98*	1										
EC	67,706	1.3 (0.2)	1.2	1.3	1.4	0.22	−0.01	0.87*	0.76*	1									
Nitrate	67,706	1.4 (0.8)	0.6	1.3	2.1	1.48	−0.32*	0.57*	0.58*	0.43*	1								
Sulphate	67,706	2.8 (1.0)	1.9	2.8	3.6	1.77	0.88*	−0.42*	−0.44*	−0.31*	−0.41*	1							
Ammonium	67,706	1.5 (0.3)	1.2	1.5	1.7	0.56	0.70*	0.00	-0.02*	0.04*	0.32*	0.70*	1						
Chloride	67,706	0.20 (0.14)	0.08	0.15	0.34	0.26	−0.60*	0.69*	0.70*	0.53*	0.84*	−0.72*	−0.07*	1					
Sodium	67,706	0.15 (0.03)	0.12	0.15	0.17	0.06	0.52*	−0.32*	−0.31*	−0.29*	−0.72*	0.59*	0.04*	−0.80*	1				
Potassium	67,706	0.07 (0.01)	0.06	0.07	0.08	0.02	0.06*	0.65*	0.61*	0.64*	0.24*	−0.15*	0.04*	0.22*	0.18*	1			
Calcium	67,706	0.07 (0.02)	0.06	0.07	0.09	0.03	0.37*	−0.12*	−0.17*	0.04*	0.17*	0.43*	0.58*	−0.14*	0.18*	0.40*	1		
Ozone (ppb)^b^	83,454	35.9 (7.4)	29.3	35.0	42.7	13.41	0.83*	−0.46*	−0.45*	−0.41*	−0.41*	0.86*	0.59*	−0.75*	0.65*	−0.02*	0.50*	1	
Ambient temperature (°C)	83,454	16.5 (7.0)	9.7	16.7	23.3	13.54	0.55*	−0.36*	−0.39*	−0.23*	−0.87*	0.57*	−0.08*	−0.85*	0.86*	−0.08*	−0.12*	0.52*	1

*EC* elemental carbon, *IQR* interquartile range, *OC* organic carbon, *SD* standard deviation.

*p value <0.05.

^a^Exposure to PM_2.5_ chemical components was assigned to 67,706 women, because these measurements only began in April 2013, which the study began in January of that year.

^b^Daily maximum 8-h mean concentrations.

The association between pollutants exposure over the first trimester and placenta-mediated pregnancy complications is shown in Table 3. Total PM_2.5_ and ozone were not significantly associated with a composite outcome. Compared with the lowest group, however, the point estimates of the OR in the highest group were above unity (OR for PM_2.5_ = 1.07, 95% CI = 0.95–1.19; for ozone 1.12, 0.98–1.28). With regard to PM_2.5_ chemical components, total carbon, particularly OC and sodium were positively associated with outcome. The ORs per IQR increase in the concentrations of carbon (IQR = 0.62 μg/m^3^), OC (0.51 μg/m^3^) and sodium (0.06 μg/m^3^) were 1.10 (95% CI = 1.04–1.17), 1.15 (1.08–1.22) and 1.11 (1.03–1.18), respectively. Pollutant exposure over the 3 months before pregnancy was not associated with outcome (Table S1). With respect to exposure over the second trimester, there was no evidence of increased odds of outcome, in fact, decreased odds were observed with increasing concentrations of some components (Table S2).

**Table 3:** Odds ratios (ORs) and 95% confidence intervals (CIs) for the association between exposure to PM_2.5_ and ozone over the first trimester (0–13 weeks of gestation) and placenta-mediated pregnancy complications.

	Quintile											
	1 (lowest)		2		3		4		5 (highest)		Per IQR increase	
Total PM_2.5_ (μg/m^3^)												
Median (IQR)	14.0 (13.1–14.3)		15.2 (14.9–15.5)		16.1 (15.8–16.4)		17.8 (17.5–18.5)		21.3 (20.5–21.7)			
No. of women	16,616		16,736		16,678		16,714		16,710		83,454	
No. of outcome	1669		1792		1761		1783		1658		8663	
OR (95% CI)^a^	Reference		1.09 (1.01–1.18)		1.11 (1.02–1.22)		1.12 (1.01–1.25)		1.07 (0.96–1.20)		1.00 (0.94–1.05)	
OR (95% CI)^b^	Reference		1.09 (1.01–1.18)		1.11 (1.02–1.22)		1.13 (1.02–1.25)		1.07 (0.96–1.19)		0.99 (0.94–1.05)	
PM_2.5_ components (μg/m^3^)												
Total carbon												
Median (IQR)	3.2 (2.8–3.5)		3.7 (3.7–3.8)		4.1 (4.0–4.1)		4.2 (4.2–4.3)		4.6 (4.4–4.9)			
No. of women	13,508		13,492		13,584		13,569		13,553		67,706	
No. of outcome	1403		1358		1433		1385		1479		7058	
OR (95% CI)^a^	Reference		0.96 (0.87–1.06)		1.10 (0.98–1.22)		1.06 (0.95–1.18)		1.11 (0.98–1.25)		1.10 (1.04–1.17)	
OR (95% CI)^b^	Reference		0.96 (0.87–1.06)		1.10 (0.98–1.23)		1.06 (0.95–1.19)		1.11 (0.98–1.26)		1.10 (1.04–1.17)	
OC												
Median (IQR)	2.1 (1.8–2.3)		2.4 (2.4–2.5)		2.7 (2.7–2.8)		2.9 (2.8–2.9)		3.2 (3.0–3.3)			
No. of women	13,540		13,486		13,569		13,526		13,585		67,706	
No. of outcome	1398		1351		1379		1402		1528		7058	
OR (95% CI)^a^	Reference		0.97 (0.87–1.07)		1.07 (0.97–1.19)		1.10 (0.98–1.22)		1.19 (1.05–1.34)		1.14 (1.08–1.22)	
OR (95% CI)^b^	Reference		0.97 (0.87–1.07)		1.08 (0.97–1.20)		1.10 (0.99–1.23)		1.20 (1.06–1.36)		1.15 (1.08–1.22)	
EC												
Median (IQR)	1.1 (1.0–1.2)		1.2 (1.2–1.3)		1.3 (1.3–1.3)		1.4 (1.4–1.4)		1.5 (1.5–1.5)			
No. of women	13,483		13,549		13,543		13,547		13,584		67,706	
No. of outcome	1440		1452		1373		1365		1428		7058	
OR (95% CI)^a^	Reference		1.00 (0.91–1.11)		0.91 (0.83–1.01)		0.94 (0.86–1.04)		0.99 (0.89–1.10)		1.01 (0.96–1.07)	
OR (95% CI)^b^	Reference		1.00 (0.91–1.10)		0.91 (0.82–1.00)		0.94 (0.85–1.03)		0.98 (0.89–1.09)		1.01 (0.96–1.07)	
Nitrate												
Median (IQR)	0.3 (0.2–0.4)		0.7 (0.6–0.8)		1.3 (1.1–1.5)		1.9 (1.8–2.1)		2.6 (2.5–2.7)			
No. of women	13,502		13,547		13,537		13,539		13,581		67,706	
No. of outcome	1416		1424		1424		1414		1380		7058	
OR (95% CI)^a^	Reference		0.96 (0.88–1.05)		1.01 (0.91–1.12)		1.02 (0.91–1.15)		1.02 (0.90–1.17)		1.03 (0.95–1.13)	
OR (95% CI)^b^	Reference		0.96 (0.88–1.05)		1.02 (0.92–1.13)		1.03 (0.91–1.16)		1.03 (0.90–1.17)		1.03 (0.95–1.13)	
Sulphate												
Median (IQR)	1.7 (1.5–1.7)		2.0 (1.9–2.1)		2.8 (2.5–2.9)		3.4 (3.2–3.6)		4.1 (3.9–4.8)			
No. of women	13,528		13,481		13,547		13,584		13,566		67,706	
No. of outcome	1442		1405		1380		1443		1388		7058	
OR (95% CI)^a^	Reference		1.00 (0.91–1.11)		1.01 (0.89–1.14)		1.10 (0.96–1.25)		1.10 (0.95–1.28)		1.05 (0.97–1.13)	
OR (95% CI)^b^	Reference		1.00 (0.91–1.11)		1.01 (0.89–1.15)		1.10 (0.96–1.26)		1.10 (0.95–1.28)		1.05 (0.97–1.13)	
Ammonium												
Median (IQR)	1.0 (1.0–1.1)		1.2 (1.2–1.3)		1.5 (1.4–1.6)		1.7 (1.7–1.7)		1.8 (1.8–1.9)			
No. of women	13,452		13,536		13,558		13,611		13,549		67,706	
No. of outcome	1447		1436		1417		1393		1365		7058	
OR (95% CI)^a^	Reference		0.98 (0.90–1.06)		1.02 (0.93–1.11)		1.03 (0.93–1.15)		1.03 (0.92–1.16)		1.03 (0.95–1.12)	
OR (95% CI)^b^	Reference		0.98 (0.90–1.06)		1.01 (0.93–1.11)		1.03 (0.93–1.15)		1.03 (0.92–1.16)		1.03 (0.95–1.12)	
Chloride												
Median (IQR)	0.04 (0.03–0.06)		0.09 (0.08–0.11)		0.15 (0.14–0.18)		0.30 (0.26–0.34)		0.41 (0.39–0.44)			
No. of women	13,531		13,497		13,571		13,508		13,599		67,706	
No. of outcome	1379		1404		1412		1441		1422		7058	
OR (95% CI)^a^	Reference		1.00 (0.92–1.09)		1.06 (0.96–1.17)		1.16 (1.01–1.33)		1.11 (0.96–1.29)		1.04 (0.95–1.14)	
OR (95% CI)^b^	Reference		1.00 (0.92–1.09)		1.06 (0.97–1.17)		1.16 (1.01–1.33)		1.11 (0.96–1.28)		1.04 (0.95–1.14)	
Sodium												
Median (IQR)	0.10 (0.09–0.10)		0.13 (0.12–0.13)		0.15 (0.15–0.16)		0.17 (0.16–0.17)		0.19 (0.18–0.19)			
No. of women	13,516		13,546		13,561		13,499		13,584		67,706	
No. of outcome	1388		1391		1415		1365		1499		7058	
OR (95% CI)^a^	Reference		1.02 (0.94–1.10)		1.08 (0.98–1.18)		1.02 (0.92–1.14)		1.15 (1.02–1.29)		1.10 (1.03–1.18)	
OR (95% CI)^b^	Reference		1.02 (0.94–1.11)		1.08 (0.98–1.19)		1.03 (0.93–1.15)		1.16 (1.03–1.31)		1.11 (1.03–1.18)	
Potassium												
Median (IQR)	0.06 (0.05–0.06)		0.06 (0.06–0.06)		0.07 (0.06–0.07)		0.08 (0.07–0.08)		0.09 (0.09–0.10)			
No. of women	13,483		13,590		13,510		13,473		13,650		67,706	
No. of outcome	1409		1354		1404		1425		1466		7058	
OR (95% CI)^a^	Reference		0.95 (0.88–1.04)		0.99 (0.91–1.08)		1.04 (0.95–1.14)		1.03 (0.94–1.12)		1.05 (1.00–1.09)	
OR (95% CI)^b^	Reference		0.95 (0.88–1.03)		0.98 (0.90–1.07)		1.04 (0.95–1.13)		1.03 (0.94–1.12)		1.05 (1.00–1.09)	
Calcium												
Median (IQR)	0.05 (0.04–0.05)		0.06 (0.06–0.07)		0.07 (0.07–0.07)		0.09 (0.08–0.09)		0.11 (0.10–0.11)			
No. of women	13,507		13,574		13,496		13,539		13,590		67,706	
No. of outcome	1411		1389		1490		1407		1361		7058	
OR (95% CI)^a^	Reference		0.96 (0.88–1.04)		1.04 (0.95–1.13)		1.02 (0.93–1.12)		1.02 (0.92–1.13)		1.02 (0.97–1.07)	
OR (95% CI)^b^	Reference		0.95 (0.87–1.04)		1.04 (0.96–1.13)		1.02 (0.93–1.12)		1.02 (0.92–1.13)		1.01 (0.97–1.07)	
Ozone (ppb)												
Median (IQR)	25.8 (25.0–26.5)		30.3 (28.8–31.4)		33.8 (32.9–36.0)		40.4 (38.5–42.1)		45.4 (44.1–47.8)			
No. of women	16,669		16,700		16,670		16,707		16,708		83,454	
No. of outcome	1731		1732		1746		1716		1738		8663	
OR (95% CI)^a^	Reference		1.02 (0.93–1.13)		1.04 (0.92–1.17)		1.08 (0.96–1.23)		1.12 (0.98–1.28)		1.05 (0.97–1.14)	
OR (95% CI)^b^	Reference		1.02 (0.93–1.13)		1.04 (0.92–1.18)		1.09 (0.96–1.23)		1.12 (0.98–1.28)		1.05 (0.97–1.14)	

*EC* elemental carbon, *IQR* interquartile range, *OC* organic carbon.

^a^Adjusted for maternal age, birth year, season of conception.

^b^Additionally adjusted for smoking, alcohol drinking, prepregnancy body mass index, current history of diabetes/gestational diabetes, infertility treatment and parity.

**Fig. 1:**
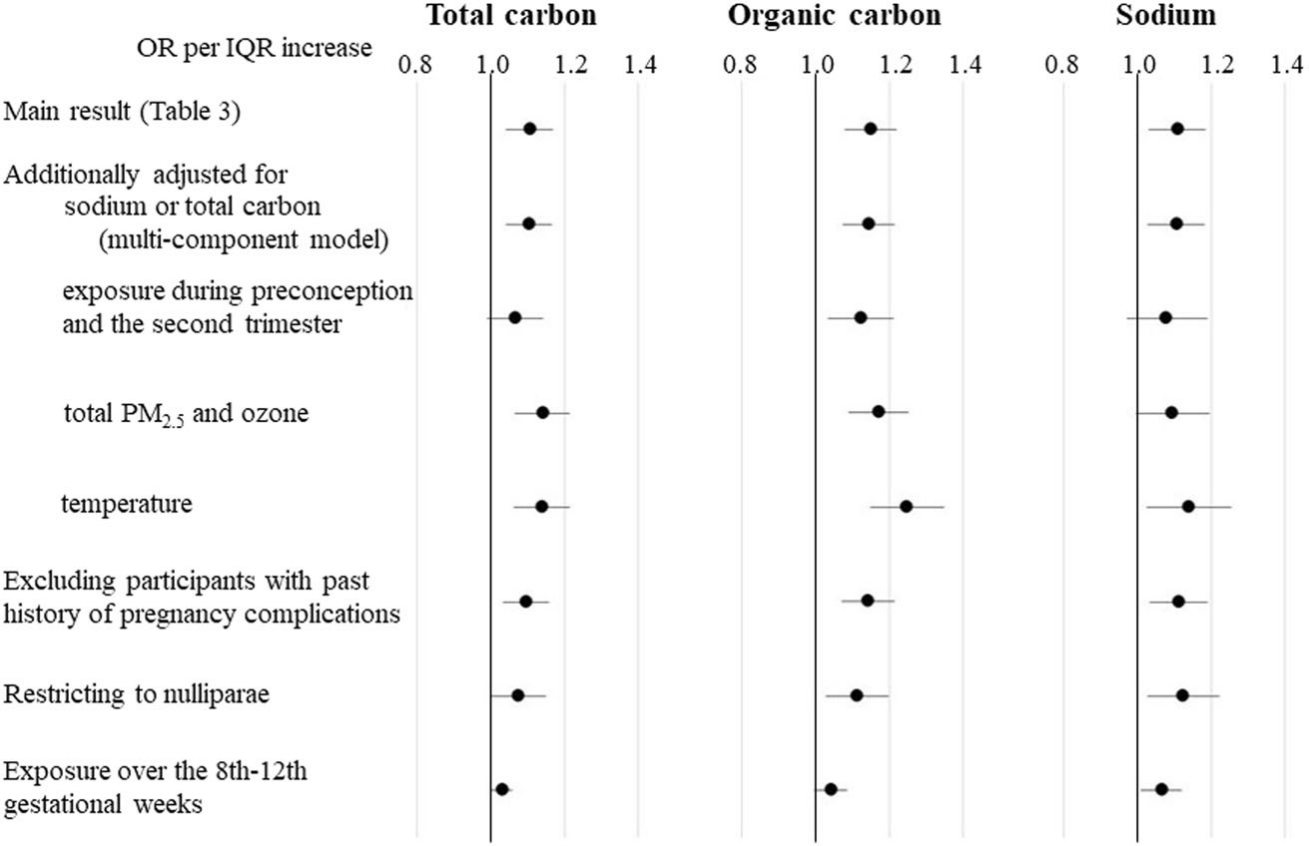
Sensitivity analysis of the association between exposure to carbon and sodium over the first trimester (0–13 weeks of gestation) and placenta-mediated pregnancy complications. The odds ratios (ORs) per interquartile range increase in the concentrations of each component were adjusted for maternal age, birth year, season of conception, smoking, alcohol drinking, prepregnancy body mass index, current history of diabetes/gestational diabetes, infertility treatment and parity. The error bars indicate 95% confidence intervals.

We investigated in detail the association of carbon and sodium with placenta-mediated pregnancy complications (Fig. 1). Overall, the tendency towards a positive association persisted. For example, the multi-component model suggested that carbon and sodium were independently associated with outcome. Also, exposure to these components from 8 to 12 weeks was positively associated with outcome.

The respective associations for individual outcomes of placenta-mediated pregnancy complications are summarised in Tables S3. In particularly, we observed that OC was consistently associated with all four complications; the ORs per IQR increase were 1.10 (95% CI = 1.02–1.17) for SGA, 1.16 (0.99–1.37) for preeclampsia, 1.29 (1.11–1.51) for placental abruption and 2.04 (1.49–2.77) for stillbirth.

## Discussion

In the analysis of singleton pregnant women in Tokyo between 2013 and 2015, exposure to total PM_2.5_ was not associated with placenta-mediated pregnancy complications (mixed outcome of SGA, preeclampsia, placental abruption and stillbirth). In the latest report by the US Environmental Protection Agency, the evidence for the association between maternal exposure to total PM_2.5_ and pregnancy and birth outcomes, including SGA, preeclampsia and stillbirth, was not consistent [9]. In contrast, we here observed that an increase in the concentrations of some PM_2.5_ chemical components, such as total carbon and sodium, was clearly associated with an elevated occurrence of pregnancy complications. This positive association was observed for exposure over the first trimester, but not for exposure over the 3 months before pregnancy or over the second trimester.

After adjustment for total PM_2.5_, we found that exposure to total carbon was positively associated with placenta-mediated pregnancy complications. This result suggested that carbon-containing components of PM_2.5_ may have contributed to pregnancy complications. Carbon is a major component in PM_2.5_ mass concentrations [25]. Some studies in the USA and Canada based on registry data reported that prenatal exposure to total carbon, mainly EC (or black carbon), was associated with the risk of SGA [26, 27]. Further, analysis based on similar data in Florida between 2004 and 2007 suggested that exposure to EC over the first trimester (mean = 0.5 μg/m^3^) increased the risk of preeclampsia (OR per IQR = 1.08, 95% CI = 1.01–1.16) and placental abruption (1.38, 1.09–1.75) [3, 4]. This study did not include exposure to OC. Past studies suggested that, among the total carbon components, EC rather than OC seems likely to be associated with adverse perinatal health. In our study, however, OC was consistently associated not only with a composite outcome but also with individual complications. EC is a primary pollutant due to incomplete combustion of fossil fuels and biomass [28], whereas OC has both primary and secondary origins [25]. However, though they differ in source origins, the concentrations of EC and OC are well correlated, and these are complex mixtures, including, for example, polycyclic aromatic hydrocarbons (PAHs). PAHs can cross the placental barrier, and are possible pollutants that affect maternal and child health [29]. Therefore, the association we observed would not be competing with past evidence regarding perinatal health effects of carbon-containing components of PM_2.5_. We also found a positive association between exposure to sodium and outcome. A few studies found the adverse perinatal health effects from sodium [30]. Although sodium is an indicator component of PM_2.5_ that originates from sea salt [31], other sea salt-related components, such as chloride, were not associated with the outcome. In this study, we did not observe the adverse effects from nitrate, sulphate or ammonium, which are markers of secondary inorganic aerosols as a major source of PM_2.5_ [31]. As there is limited evidence regarding the association between PM_2.5_ components and placenta-mediated pregnancy complications, further studies are required.

Our findings suggested that the first trimester was susceptible to pollutants exposure associated with placenta-mediated pregnancy complications. The first trimester of pregnancy, especially the latter half, is a crucial period in placentation [11]. Abnormal placentation and failure of trophoblast invasion into the placental bed are considered to be involved in the pathogenesis of preeclampsia, and to lead to foetal growth restriction related to SGA [11, 32]. Further, it has been noted that impaired placentation is the basis for the development of placental abruption [33]. Inflammation, hypoxia and coagulation in the intrauterine environment appears to contribute to the pathophysiology of abnormal placental development [12, 32]. These biological reactions are on the biological pathway of exposure to PM_2.5_ [34], and short-term exposure to OC was associated with an elevated level of inflammatory biomarkers, such as tumour necrosis factor alpha, in healthy adults [35]. Furthermore, a birth cohort study in Canada reported that hyperhomocysteinemia, which is related to inflammation and increased coagulation [36], was associated with an elevated risk of placenta-mediated pregnancy complications [37]. Plasma levels of homocysteine were higher in taxi drivers, who were routinely exposed to air pollutants, such as PAHs as carbonaceous constituent of PM_2.5_, than in non-occupationally exposed individuals [38]. Therefore, it is likely that first-trimester exposure to pollutants, such as OC, has a role in abnormal placentation. Incidentally, the OR point estimates for the association with exposure to some components over the second trimester showed below unity. Since we did not have reason to believe that the direction of this association was biologically plausible, we interpreted this to mean that second trimester exposure had little influence on pregnancy complications.

We previously reported a positive association between exposure to ozone during the first trimester and preeclampsia in 33,380 singleton pregnant women who resided in western Japan between 2005 and 2010 [39]. In the present study, there was no significant association (Table S3). This difference might partially reflect different ozone concentrations, so those in the previous study (mean = 41.3 ppb) tended to be higher than those in the present study (35.3 ppb).

Some methodological issues are worth mentioning. One clear limitation is that we did not consider spatial variability in PM_2.5_ concentrations within the 23 Tokyo wards; we assessed only temporal variability in these concentrations. In environmental epidemiological study, however, pollutant concentrations measured at the central monitoring station are often assigned to all participants living within a certain distance from the station. In this case, the area of the 23 Tokyo wards can be covered with a buffer size of roughly 20 km (Fig. S1), and a California study reported that the association between PM_2.5_ components and birth weight with a 20-km buffer was similar to that with a 10-km buffer [30]. We considered, therefore, that homogeneous pollutant concentrations within the 23 Tokyo wards was an acceptable assumption. However, it is possible that this spatial homogeneous assumption would not apply in the case of some of the components. Also, we assumed that the temporal variation was similar for all components within the 23 studied wards. Thus, we should interpret the respective associations for individual components cautiously. In addition, we did not adjust for heterogeneous spatial pollutants (such as nitrogen dioxide) as co-pollutants [40]. Second, we did not have information on residential mobility during pregnancy. Even after we excluded *‘satogaeri’* cases, women who resided far away from the study area in early pregnancy might have been included in the study population. However, a review suggested that most moves during pregnancy did not significantly affect exposure estimates, as most were intra-neighbourhood moves, though the percentage of women who moved during pregnancy was not negligible (median = 20%) [41]. Third, we did not have information on socioeconomic status as a possible confounding factor [42]. However, by using a multilevel model with the hospital as a random effect, we considered a hospital catchment area that likely had a relatively uniform socioeconomic status. Finally, we had a weakness in the generalisability of our results, as the Perinatal Registry database was established mainly with information from university and local general hospitals.

Irrespective of these limitations, to the best of our knowledge, this is the first study focused on the association between exposure to PM_2.5_ chemical components over the first trimester and placenta-mediated pregnancy complications as a composite outcome. Another strength is that we used continuous filter-based measurements of PM_2.5_ components in a megacity, Tokyo, and this allowed us to analyse a relatively large sample size. In addition, we analysed data derived from a good-quality clinical database, including information on major confounding factors related to placenta-mediated pregnancy complications, such as smoking habits, prepregnancy overweight, and past history of pregnancy complications.

In conclusion, we found that exposure to some components of PM_2.5_ over the first trimester was positively associated with placenta-mediated pregnancy complications in Tokyo, a highly urbanised major global city. Our findings suggest that specific components of PM_2.5_ have harmful effects on placentation in urbanised settings.

## Supplementary information

Supplementary information

Supplementary materials
