# Supplementary material for: Exposure to chemical components of fine particulate matter and ozone, and placenta-mediated pregnancy complications in Tokyo: a register-based study
Source: J Expo Sci Environ Epidemiol. 2021 Feb 18;32(1):135–45. doi: 10.1038/s41370-021-00299-4 (PMC8770113; doi:10.1038/s41370-021-00299-4)
Supplement: Supplementary file 1 — Supplementary information [file 41370_2021_299_MOESM1_ESM.docx]

**Supplementary information**

**Table S1** Odds ratios (ORs) and 95% confidence intervals (CIs) for the association between exposure to PM_2.5_ and ozone over the three months before pregnancy and placenta-mediated pregnancy complications.

**Table S2** Odds ratios (ORs) and 95% confidence intervals (CIs) for the association between exposure to PM_2.5_ and ozone over the second trimester (14-27 weeks of gestation) and placenta-mediated pregnancy complications.

**Table S3** Association between exposure to PM_2.5_ and ozone over the first trimester (0-13 weeks of gestation) and individual outcomes of placenta-mediated pregnancy complications

**Fig. S1** Locations of monitoring stations and hospitals.
