# Supplementary material for: Exposure to chemical components of fine particulate matter and ozone, and placenta-mediated pregnancy complications in Tokyo: a register-based study
Source: J Expo Sci Environ Epidemiol. 2021 Feb 18;32(1):135–45. doi: 10.1038/s41370-021-00299-4 (PMC8770113; doi:10.1038/s41370-021-00299-4)
Supplement: Supplementary file 2 — Supplementary materials [file 41370_2021_299_MOESM2_ESM.doc]

**Supplementary materials**

**Exposure to chemical components of fine particulate matter and ozone, and placenta- mediated pregnancy complications in Tokyo: A register-based study**

Takehiro Michikawa^1^・Seiichi Morokuma^2^・Shin Yamazaki^3^・Akinori Takami^4^・Seiji Sugata^4^・ Ayako Yoshino^4^・Yuki Takeda^1^・Kazushige Nakahara^5^・Shinji Saito^6^・Junya Hoshi^6^・Kiyoko Kato^5^・Hiroshi Nitta^3^・Yuji Nishiwaki^1^

^1^ Department of Environmental and Occupational Health, School of Medicine, Toho

University, 5-21-16 Omori-nishi, Ota-ku, Tokyo 143-8540, Japan

^2^ Department of Health Sciences, Graduate School of Medical Sciences, Kyushu University, 3-1-1 Maidashi, Higashi-ku, Fukuoka 812-8582, Japan

^3^ Centre for Health and Environmental Risk Research, National Institute for Environmental Studies, 16-2 Onogawa, Tsukuba, Ibaraki 305-8506, Japan

^4^ Centre for Regional Environmental Research, National Institute for Environmental Studies, 16-2 Onogawa, Tsukuba, Ibaraki 305-8506, Japan

^5^ Department of Obstetrics and Gynaecology, Graduate School of Medical Sciences, Kyushu University, 3-1-1 Maidashi, Higashi-ku, Fukuoka 812-8582, Japan

^6^ Tokyo Metropolitan Research Institute for Environmental Protection, 1-7-5 Shinsuna, Koto-ku, Tokyo 136-0075, Japan

**Correspondence:**

Takehiro Michikawa, MD, PhD

Department of Environmental and Occupational Health,

School of Medicine, Toho University,

5-21-16 Omori-nishi, Ota-ku, Tokyo 143-8540, Japan.

TEL: 81-3-3762-4151 ext.2405

E-mail: [takehiro.michikawa@med.toho-u.ac.jp](mailto:takehiro.michikawa@med.toho-u.ac.jp)

**Table of contents:**

Table S1……....……………………………………………………………………………… 2

Table S2……....……………………………………………………………………………… 4

Table S3……....……………………………………………………………………………… 6

Fig. S1…………………...…………………………………………………………………… 8

**Table S1** 　Odds ratios (ORs) and 95% confidence intervals (CIs) for the association between exposure to PM_2.5_ and ozone over the three months before pregnancy and placenta-mediated pregnancy complications.

|  |  |  |  | Quintile | | | | | | | | | |  |  |  |  |
| --- | --- | --- | --- | --- | --- | --- | --- | --- | --- | --- | --- | --- | --- | --- | --- | --- | --- |
|  |  |  |  | 1 (lowest) | | 2 | | 3 | | 4 | | 5 (highest) | |  | per IQR increase | | |
| Total PM_2.5_ (μg/m^3^) | | | Median (IQR) | 13.5 | (13.1-14.3) | 14.9 | (14.7-15.1) | 15.7 | (15.6-16.0) | 17.6 | (16.9-18.1) | 21.8 | (20.5-22.3) |  |  |  |  |
|  |  |  | No. of women | 16,668 | | 16,642 | | 16,679 | | 16,728 | | 16,737 | |  | 83,454 | | |
|  |  |  | No. of outcome | 1,648 | | 1,746 | | 1,717 | | 1,813 | | 1,739 | |  | 8,663 | | |
|  |  |  | OR (95% CI)^a^ | Reference | | 1.10 | (1.02-1.19) | 1.04 | (0.96-1.14) | 1.14 | (1.03-1.26) | 1.04 | (0.93-1.17) |  | 1.00 | (0.96-1.05) | |
| PM_2.5_ components (μg/m^3^) | | | |  | | | | | | | | | | | | | |
|  | Total carbon | | Median (IQR) | 2.9 | (2.8-3.3) | 3.6 | (3.6-3.7) | 4.0 | (3.8-4.1) | 4.2 | (4.2-4.3) | 4.7 | (4.4-5.0) |  |  |  |  |
|  |  |  | No. of women | 12,209 | | 12,442 | | 12,370 | | 12,296 | | 12,413 | |  | 61,730 | | |
|  |  |  | No. of outcome | 1,266 | | 1,332 | | 1,321 | | 1,246 | | 1,294 | |  | 6,459 | | |
|  |  |  | OR (95% CI)^a^ | Reference | | 0.99 | (0.90-1.09) | 1.04 | (0.94-1.14) | 0.96 | (0.86-1.07) | 0.96 | (0.84-1.10) |  | 0.99 | (0.95-1.04) | |
|  |  | OC | Median (IQR) | 1.8 | (1.8-2.1) | 2.3 | (2.3-2.4) | 2.6 | (2.5-2.7) | 2.8 | (2.8-2.9) | 3.2 | (3.0-3.4) |  |  |  |  |
|  |  |  | No. of women | 12,269 | | 12,409 | | 12,346 | | 12,278 | | 12,428 | |  | 61,730 | | |
|  |  |  | No. of outcome | 1,291 | | 1,323 | | 1,269 | | 1,262 | | 1,314 | |  | 6,459 | | |
|  |  |  | OR (95% CI)^a^ | Reference | | 0.98 | (0.90-1.08) | 0.99 | (0.90-1.09) | 0.98 | (0.88-1.09) | 1.00 | (0.88-1.15) |  | 1.01 | (0.96-1.06) | |
|  |  | EC | Median (IQR) | 1.0 | (1.0-1.1) | 1.3 | (1.2-1.3) | 1.3 | (1.3-1.3) | 1.4 | (1.4-1.4) | 1.5 | (1.5-1.6) |  |  |  |  |
|  |  |  | No. of women | 12,260 | | 12,163 | | 12,614 | | 12,325 | | 12,368 | |  | 61,730 | | |
|  |  |  | No. of outcome | 1,277 | | 1,350 | | 1,299 | | 1,268 | | 1,265 | |  | 6,459 | | |
|  |  |  | OR (95% CI)^a^ | Reference | | 1.05 | (0.95-1.15) | 0.94 | (0.85-1.04) | 0.96 | (0.87-1.07) | 0.92 | (0.82-1.03) |  | 0.96 | (0.92-1.01) | |
|  | Nitrate | | Median (IQR) | 0.2 | (0.2-0.3) | 0.6 | (0.4-0.7) | 1.0 | (0.9-1.2) | 1.7 | (1.6-1.8) | 2.6 | (2.3-2.7) |  |  |  |  |
|  |  |  | No. of women | 12,329 | | 12,286 | | 12,390 | | 12,323 | | 12,402 | |  | 61,730 | | |
|  |  |  | No. of outcome | 1,302 | | 1,321 | | 1,272 | | 1,273 | | 1,291 | |  | 6,459 | | |
|  |  |  | OR (95% CI)^a^ | Reference | | 1.00 | (0.92-1.10) | 0.92 | (0.83-1.01) | 0.94 | (0.83-1.07) | 1.03 | (0.91-1.17) |  | 1.01 | (0.93-1.09) | |
|  | Sulphate | | Median (IQR) | 1.6 | (1.4-1.7) | 1.9 | (1.8-2.0) | 2.6 | (2.3-2.9) | 3.3 | (3.2-3.6) | 4.3 | (4.1-5.1) |  |  |  |  |
|  |  |  | No. of women | 12,288 | | 12,305 | | 12,423 | | 12,349 | | 12,365 | |  | 61,730 | | |
|  |  |  | No. of outcome | 1,314 | | 1,233 | | 1,343 | | 1,270 | | 1,299 | |  | 6,459 | | |
|  |  |  | OR (95% CI)^a^ | Reference | | 0.93 | (0.85-1.02) | 1.01 | (0.90-1.14) | 0.95 | (0.84-1.08) | 0.95 | (0.83-1.08) |  | 0.98 | (0.92-1.04) | |
|  | Ammonium | | Median (IQR) | 1.0 | (0.9-1.1) | 1.2 | (1.1-1.2) | 1.4 | (1.3-1.4) | 1.7 | (1.6-1.8) | 1.9 | (1.8-1.9) |  |  |  |  |
|  |  |  | No. of women | 12,164 | | 12,443 | | 12,362 | | 12,391 | | 12,370 | |  | 61,730 | | |
|  |  |  | No. of outcome | 1,275 | | 1,334 | | 1,261 | | 1,271 | | 1,318 | |  | 6,459 | | |
|  |  |  | OR (95% CI)^a^ | Reference | | 1.03 | (0.94-1.13) | 1.01 | (0.92-1.11) | 1.00 | (0.90-1.10) | 1.02 | (0.92-1.14) |  | 1.00 | (0.94-1.07) | |
|  | Chloride | | Median (IQR) | 0.04 | (0.02-0.05) | 0.08 | (0.08-0.09) | 0.14 | (0.12-0.15) | 0.26 | (0.21-0.33) | 0.44 | (0.41-0.46) |  |  |  |  |
|  |  |  | No. of women | 12,305 | | 12,341 | | 12,366 | | 12,351 | | 12,367 | |  | 61,730 | | |
|  |  |  | No. of outcome | 1,282 | | 1,332 | | 1,297 | | 1,258 | | 1,290 | |  | 6,459 | | |
|  |  |  | OR (95% CI)^a^ | Reference | | 0.97 | (0.88-1.07) | 0.93 | (0.84-1.04) | 0.94 | (0.82-1.06) | 0.98 | (0.85-1.12) |  | 1.04 | (0.96-1.13) | |
|  | Sodium | | Median (IQR) | 0.10 | (0.09-0.10) | 0.13 | (0.12-0.14) | 0.15 | (0.15-0.16) | 0.16 | (0.16-0.17) | 0.19 | (0.18-0.20) |  |  |  |  |
|  |  |  | No. of women | 12,345 | | 12,302 | | 12,389 | | 12,326 | | 12,368 | |  | 61,730 | | |
|  |  |  | No. of outcome | 1,285 | | 1,260 | | 1,292 | | 1,260 | | 1,362 | |  | 6,459 | | |
|  |  |  | OR (95% CI)^a^ | Reference | | 0.98 | (0.90-1.07) | 0.96 | (0.87-1.06) | 0.96 | (0.85-1.08) | 1.03 | (0.91-1.17) |  | 1.03 | (0.96-1.10) | |
|  | Potassium | | Median (IQR) | 0.05 | (0.05-0.05) | 0.06 | (0.06-0.06) | 0.06 | (0.06-0.07) | 0.07 | (0.07-0.08) | 0.09 | (0.09-0.10) |  |  |  |  |
|  |  |  | No. of women | 12,322 | | 12,340 | | 12,353 | | 12,282 | | 12,433 | |  | 61,730 | | |
|  |  |  | No. of outcome | 1,292 | | 1,267 | | 1,306 | | 1,302 | | 1,292 | |  | 6,459 | | |
|  |  |  | OR (95% CI)^a^ | Reference | | 1.00 | (0.91-1.08) | 1.02 | (0.93-1.11) | 0.99 | (0.90-1.08) | 0.96 | (0.87-1.06) |  | 0.97 | (0.93-1.01) | |
|  | Calcium | | Median (IQR) | 0.04 | (0.04-0.05) | 0.06 | (0.06-0.06) | 0.07 | (0.07-0.07) | 0.08 | (0.08-0.09) | 0.11 | (0.10-0.11) |  |  |  |  |
|  |  |  | No. of women | 12,312 | | 12,323 | | 12,381 | | 12,367 | | 12,347 | |  | 61,730 | | |
|  |  |  | No. of outcome | 1,275 | | 1,365 | | 1,280 | | 1,298 | | 1,241 | |  | 6,459 | | |
|  |  |  | OR (95% CI)^a^ | Reference | | 1.04 | (0.95-1.13) | 0.96 | (0.88-1.05) | 0.98 | (0.89-1.07) | 0.95 | (0.86-1.02) |  | 0.98 | (0.93-1.02) | |
| Ozone (ppb) | | | Median (IQR) | 25.7 | (25.0-26.3) | 30.7 | (29.1-31.8) | 34.6 | (33.5-36.9) | 41.5 | (39.8-42.9) | 46.0 | (44.4-47.2) |  |  |  |  |
|  |  |  | No. of women | 16,637 | | 16,709 | | 16,718 | | 16,667 | | 16,723 | |  | 83,454 | | |
|  |  |  | No. of outcome | 1,696 | | 1,682 | | 1,796 | | 1,747 | | 1,742 | |  | 8,663 | | |
|  |  |  | OR (95% CI)^a^ | Reference | | 0.97 | (0.88-1.08) | 1.03 | (0.90-1.16) | 1.01 | (0.89-1.15) | 0.97 | (0.85-1.11) |  | 1.00 | (0.93-1.08) | |

*EC* elemental carbon, *IQR* interquartile range, *OC* organic carbon.

^a^Adjusted for maternal age, birth year, season of conception, smoking, alcohol drinking, prepregnancy body mass index, current history of diabetes/gestational diabetes, infertility treatment, and parity.

**Table S2** Odds ratios (ORs) and 95% confidence intervals (CIs) for the association between exposure to PM_2.5_ and ozone over the second trimester (14-27 weeks of gestation) and placenta-mediated pregnancy complications.^a^

|  |  |  |  | Quintile | | | | | | | | | |  |  |  |  |
| --- | --- | --- | --- | --- | --- | --- | --- | --- | --- | --- | --- | --- | --- | --- | --- | --- | --- |
|  |  |  |  | 1 (lowest) | | 2 | | 3 | | 4 | | 5 (highest) | |  | per IQR increase | | |
| Total PM_2.5_ (μg/m^3^) | | | Median (IQR) | 14.0 | (13.1-14.3) | 15.3 | (14.9-15.5) | 16.4 | (15.9-16.7) | 17.8 | (17.5-18.5) | 21.3 | (20.3-21.7) |  |  |  |  |
|  |  |  | No. of women | 16,545 | | 16,546 | | 16,560 | | 16,547 | | 16,585 | |  | 82,783 | | |
|  |  |  | No. of outcome | 1,713 | | 1,700 | | 1,693 | | 1,662 | | 1,576 | |  | 8,344 | | |
|  |  |  | OR (95% CI)^b^ | Reference | | 0.96 | (0.89-1.04) | 0.95 | (0.87-1.04) | 0.95 | (0.86-1.04) | 0.92 | (0.83-1.02) |  | 0.97 | (0.92-1.01) | |
| PM_2.5_ components (μg/m^3^) | | | |  | | | | | | | | | |  |  |  |  |
|  | Total carbon | | Median (IQR) | 3.5 | (3.1-3.6) | 3.8 | (3.7-3.8) | 4.1 | (4.0-4.1) | 4.2 | (4.2-4.3) | 4.6 | (4.4-4.9) |  |  |  |  |
|  |  |  | No. of women | 13,386 | | 13,315 | | 13,528 | | 13,438 | | 13,466 | |  | 67,133 | | |
|  |  |  | No. of outcome | 1,393 | | 1,281 | | 1,388 | | 1,364 | | 1,360 | |  | 6,786 | | |
|  |  |  | OR (95% CI)^b^ | Reference | | 0.93 | (0.86-1.01) | 0.92 | (0.84-1.02) | 0.90 | (0.81-1.00) | 0.87 | (0.78-0.97) |  | 0.94 | (0.89-0.98) | |
|  |  | OC | Median (IQR) | 2.3 | (2.1-2.3) | 2.5 | (2.4-2.6) | 2.8 | (2.7-2.8) | 2.9 | (2.8-2.9) | 3.1 | (3.0-3.3) |  |  |  |  |
|  |  |  | No. of women | 13,349 | | 13,409 | | 13,469 | | 13,394 | | 13,512 | |  | 67,133 | | |
|  |  |  | No. of outcome | 1,365 | | 1,301 | | 1,350 | | 1,369 | | 1,401 | |  | 6,786 | | |
|  |  |  | OR (95% CI)^b^ | Reference | | 0.96 | (0.88-1.04) | 0.95 | (0.86-1.04) | 0.93 | (0.84-1.03) | 0.90 | (0.81-1.01) |  | 0.94 | (0.89-0.99) | |
|  |  | EC | Median (IQR) | 1.1 | (1.0-1.2) | 1.2 | (1.2-1.2) | 1.3 | (1.3-1.3) | 1.4 | (1.4-1.4) | 1.5 | (1.5-1.5) |  |  |  |  |
|  |  |  | No. of women | 13,325 | | 13,383 | | 13,522 | | 13,414 | | 13,489 | |  | 67,133 | | |
|  |  |  | No. of outcome | 1,418 | | 1,384 | | 1,338 | | 1,337 | | 1,309 | |  | 6,786 | | |
|  |  |  | OR (95% CI)^b^ | Reference | | 0.94 | (0.86-1.02) | 0.91 | (0.83-1.00) | 0.91 | (0.83-1.00) | 0.85 | (0.77-0.94) |  | 0.93 | (0.88-0.97) | |
|  | Nitrate | | Median (IQR) | 0.3 | (0.2-0.4) | 0.7 | (0.6-0.8) | 1.3 | (1.1-1.5) | 1.9 | (1.8-2.1) | 2.6 | (2.5-2.7) |  |  |  |  |
|  |  |  | No. of women | 13,383 | | 13,444 | | 13,351 | | 13,491 | | 13,464 | |  | 67,133 | | |
|  |  |  | No. of outcome | 1,316 | | 1,395 | | 1,269 | | 1,370 | | 1,436 | |  | 6,786 | | |
|  |  |  | OR (95% CI)^b^ | Reference | | 1.08 | (0.98-1.20) | 0.97 | (0.86-1.09) | 1.02 | (0.87-1.19) | 1.10 | (0.94-1.30) |  | 1.05 | (0.94-1.16) | |
|  | Sulphate | | Median (IQR) | 1.7 | (1.5-1.8) | 2.1 | (1.9-2.2) | 2.9 | (2.7-3.1) | 3.6 | (3.4-3.8) | 4.1 | (3.9-4.6) |  |  |  |  |
|  |  |  | No. of women | 13,408 | | 13,384 | | 13,452 | | 13,377 | | 13,512 | |  | 67,133 | | |
|  |  |  | No. of outcome | 1,385 | | 1,343 | | 1,395 | | 1,357 | | 1,306 | |  | 6,786 | | |
|  |  |  | OR (95% CI)^b^ | Reference | | 1.03 | (0.92-1.15) | 1.09 | (0.95-1.24) | 1.03 | (0.89-1.18) | 0.97 | (0.83-1.14) |  | 0.95 | (0.87-1.04) | |
|  | Ammonium | | Median (IQR) | 1.0 | (1.0-1.1) | 1.2 | (1.2-1.3) | 1.5 | (1.5-1.6) | 1.7 | (1.7-1.7) | 1.8 | (1.8-1.9) |  |  |  |  |
|  |  |  | No. of women | 13,316 | | 13,458 | | 13,474 | | 13,344 | | 13,541 | |  | 67,133 | | |
|  |  |  | No. of outcome | 1,333 | | 1,391 | | 1,377 | | 1,379 | | 1,306 | |  | 6,786 | | |
|  |  |  | OR (95% CI)^b^ | Reference | | 1.02 | (0.93-1.11) | 0.98 | (0.88-1.09) | 0.99 | (0.88-1.12) | 0.90 | (0.79-1.03) |  | 0.95 | (0.87-1.05) | |
|  | Chloride | | Median (IQR) | 0.05 | (0.03-0.08) | 0.10 | (0.09-0.11) | 0.15 | (0.14-0.17) | 0.29 | (0.25-0.34) | 0.41 | (0.39-0.44) |  |  |  |  |
|  |  |  | No. of women | 13,358 | | 13,485 | | 13,368 | | 13,478 | | 13,444 | |  | 67,133 | | |
|  |  |  | No. of outcome | 1,325 | | 1,367 | | 1,300 | | 1,364 | | 1,430 | |  | 6,786 | | |
|  |  |  | OR (95% CI)^b^ | Reference | | 1.01 | (0.91-1.11) | 0.94 | (0.84-1.05) | 0.93 | (0.81-1.08) | 0.98 | (0.83-1.15) |  | 1.05 | (0.95-1.17) | |
|  | Sodium | | Median (IQR) | 0.10 | (0.10-0.10) | 0.13 | (0.13-0.14) | 0.16 | (0.15-0.16) | 0.18 | (0.17-0.18) | 0.19 | (0.19-0.20) |  |  |  |  |
|  |  |  | No. of women | 13,396 | | 13,383 | | 13,443 | | 13,408 | | 13,503 | |  | 67,133 | | |
|  |  |  | No. of outcome | 1,420 | | 1,387 | | 1,319 | | 1,305 | | 1,355 | |  | 6,786 | | |
|  |  |  | OR (95% CI)^b^ | Reference | | 0.99 | (0.91-1.08) | 0.92 | (0.82-1.02) | 0.84 | (0.74-0.94) | 0.84 | (0.74-0.96) |  | 0.90 | (0.84-0.97) | |
|  | Potassium | | Median (IQR) | 0.06 | (0.05-0.06) | 0.06 | (0.06-0.06) | 0.07 | (0.07-0.07) | 0.08 | (0.08-0.08) | 0.09 | (0.09-0.09) |  |  |  |  |
|  |  |  | No. of women | 13,369 | | 13,467 | | 13,429 | | 13,369 | | 13,499 | |  | 67,133 | | |
|  |  |  | No. of outcome | 1,344 | | 1,420 | | 1,350 | | 1,382 | | 1,290 | |  | 6,786 | | |
|  |  |  | OR (95% CI)^b^ | Reference | | 1.03 | (0.95-1.13) | 1.03 | (0.94-1.13) | 1.02 | (0.93-1.12) | 0.93 | (0.85-1.01) |  | 0.94 | (0.90-0.99) | |
|  | Calcium | | Median (IQR) | 0.05 | (0.04-0.05) | 0.06 | (0.06-0.06) | 0.07 | (0.07-0.07) | 0.08 | (0.08-0.08) | 0.10 | (0.10-0.11) |  |  |  |  |
|  |  |  | No. of women | 13,353 | | 13,393 | | 13,516 | | 13,406 | | 13,465 | |  | 67,133 | | |
|  |  |  | No. of outcome | 1,423 | | 1,361 | | 1,355 | | 1,374 | | 1,273 | |  | 6,786 | | |
|  |  |  | OR (95% CI)^b^ | Reference | | 0.92 | (0.84-1.00) | 0.91 | (0.84-0.99) | 0.92 | (0.84-1.01) | 0.82 | (0.73-0.91) |  | 0.91 | (0.87-0.96) | |
| Ozone (ppb) | | | Median (IQR) | 26.2 | (25.6-26.8) | 31.2 | (29.6-32.3) | 37.4 | (35.6-38.1) | 42.6 | (41.2-43.6) | 47.2 | (45.9-48.0) |  |  |  |  |
|  |  |  | No. of women | 16,510 | | 16,579 | | 16,560 | | 16,489 | | 16,645 | |  | 82,783 | | |
|  |  |  | No. of outcome | 1,741 | | 1,640 | | 1,666 | | 1,682 | | 1,615 | |  | 8,344 | | |
|  |  |  | OR (95% CI)^b^ | Reference | | 0.92 | (0.81-1.04) | 0.92 | (0.80-1.06) | 0.96 | (0.82-1.12) | 0.89 | (0.76-1.14) |  | 0.96 | (0.88-1.04) | |

*EC* elemental carbon, *IQR* interquartile range, *OC* organic carbon.

^a^We analysed 82,783 women, after excluding those who gave preterm birth during the 22nd-27th weeks of gestation.

^b^Adjusted for maternal age, birth year, season of conception, smoking, alcohol drinking, prepregnancy body mass index, current history of diabetes/gestational diabetes, infertility treatment, and parity.

**Table S3** Association between exposure to PM_2.5_ and ozone over the first trimester (0-13 weeks of gestation) and individual outcomes of placenta-mediated pregnancy complications.

|  |  |  |  |  | Small for gestational age | |  | Preeclampsia | |  | Placental abruption | |  | Stillbirth | |
| --- | --- | --- | --- | --- | --- | --- | --- | --- | --- | --- | --- | --- | --- | --- | --- |
| Total PM_2.5_ (μg/m^3^) | | | No. of women |  | 81,397 | |  | 75,895 | |  | 76,099 | |  | 75,114 | |
|  |  |  | No. of outcome |  | 6,606 | |  | 1,104 | |  | 1,308 | |  | 323 | |
|  |  |  | OR per IQR increase (95% CI)^a^ |  | 0.98 | (0.93-1.04) |  | 1.00 | (0.87-1.15) |  | 1.00 | (0.87-1.14) |  | 1.17 | (0.90-1.51) |
| PM_2.5_ components (μg/m^3^) | | | |  |  |  |  |  |  |  |  |  |  |  |  |
|  | Total carbon | | No. of women |  | 66,045 | |  | 61,540 | |  | 61,727 | |  | 60,905 | |
|  |  |  | No. of outcome |  | 5,397 | |  | 892 | |  | 1,079 | |  | 257 | |
|  |  |  | OR per IQR increase (95% CI)^a^ |  | 1.07 | (1.00-1.14) |  | 1.14 | (0.98-1.32) |  | 1.19 | (1.03-1.37) |  | 1.75 | (1.33-2.30) |
|  |  | OC | No. of women |  | 66,045 | |  | 61,540 | |  | 61,727 | |  | 60,905 | |
|  |  |  | No. of outcome |  | 5,397 | |  | 892 | |  | 1,079 | |  | 257 | |
|  |  |  | OR per IQR increase (95% CI)^a^ |  | 1.10 | (1.02-1.17) |  | 1.16 | (0.99-1.37) |  | 1.29 | (1.11-1.51) |  | 2.04 | (1.49-2.77) |
|  |  | EC | No. of women |  | 66,045 | |  | 61,540 | |  | 61,727 | |  | 60,905 | |
|  |  |  | No. of outcome |  | 5,397 | |  | 892 | |  | 1,079 | |  | 257 | |
|  |  |  | OR per IQR increase (95% CI)^a^ |  | 1.00 | (0.95-1.06) |  | 1.06 | (0.92-1.22) |  | 0.99 | (0.87-1.13) |  | 1.25 | (0.96-1.64) |
|  | Nitrate | | No. of women |  | 66,045 | |  | 61,540 | |  | 61,727 | |  | 60,905 | |
|  |  |  | No. of outcome |  | 5,397 | |  | 892 | |  | 1,079 | |  | 257 | |
|  |  |  | OR per IQR increase (95% CI)^a^ |  | 1.00 | (0.90-1.10) |  | 1.06 | (0.84-1.33) |  | 1.01 | (0.81-1.26) |  | 1.60 | (1.05-2.46) |
|  | Sulphate | | No. of women |  | 66,045 | |  | 61,540 | |  | 61,727 | |  | 60,905 | |
|  |  |  | No. of outcome |  | 5,397 | |  | 892 | |  | 1,079 | |  | 257 | |
|  |  |  | OR per IQR increase (95% CI)^a^ |  | 1.02 | (0.94-1.12) |  | 0.98 | (0.88-1.21) |  | 1.15 | (0.95-1.40) |  | 1.34 | (0.94-1.92) |
|  | Ammonium | | No. of women |  | 66,045 | |  | 61,540 | |  | 61,727 | |  | 60,905 | |
|  |  |  | No. of outcome |  | 5,397 | |  | 892 | |  | 1,079 | |  | 257 | |
|  |  |  | OR per IQR increase (95% CI)^a^ |  | 0.98 | (0.89-1.08) |  | 0.99 | (0.79-1.24) |  | 1.09 | (0.88-1.35) |  | 1.58 | (1.06-2.34) |
|  | Chloride | | No. of women |  | 66,045 | |  | 61,540 | |  | 61,727 | |  | 60,905 | |
|  |  |  | No. of outcome |  | 5,397 | |  | 892 | |  | 1,079 | |  | 257 | |
|  |  |  | OR per IQR increase (95% CI)^a^ |  | 1.01 | (0.91-1.12) |  | 1.11 | (0.86-1.44) |  | 1.02 | (0.81-1.29) |  | 1.57 | (0.99-2.49) |
|  | Sodium | | No. of women |  | 66,045 | |  | 61,540 | |  | 61,727 | |  | 60,905 | |
|  |  |  | No. of outcome |  | 5,397 | |  | 892 | |  | 1,079 | |  | 257 | |
|  |  |  | OR per IQR increase (95% CI)^a^ |  | 1.07 | (0.99-1.16) |  | 1.11 | (0.92-1.35) |  | 1.26 | (1.06-1.49) |  | 1.53 | (1.08-2.17) |
|  | Potassium | | No. of women |  | 66,045 | |  | 61,540 | |  | 61,727 | |  | 60,905 | |
|  |  |  | No. of outcome |  | 5,397 | |  | 892 | |  | 1,079 | |  | 257 | |
|  |  |  | OR per IQR increase (95% CI)^a^ |  | 1.03 | (0.98-1.08) |  | 1.06 | (0.94-1.19) |  | 1.08 | (0.97-1.20) |  | 1.24 | (1.00-1.55) |
|  | Calcium | | No. of women |  | 66,045 | |  | 61,540 | |  | 61,727 | |  | 60,905 | |
|  |  |  | No. of outcome |  | 5,397 | |  | 892 | |  | 1,079 | |  | 257 | |
|  |  |  | OR per IQR increase (95% CI)^a^ |  | 1.01 | (0.95-1.07) |  | 0.97 | (0.85-1.11) |  | 1.01 | (0.89-1.14) |  | 0.95 | (0.75-1.20) |
| Ozone (ppb) | | | No. of women |  | 81,397 | |  | 75,895 | |  | 76,099 | |  | 75,114 | |
|  |  |  | No. of outcome |  | 6,606 | |  | 1,104 | |  | 1,308 | |  | 323 | |
|  |  |  | OR per IQR increase (95% CI)^a^ |  | 1.05 | (0.97-1.15) |  | 1.00 | (0.81-1.23) |  | 1.08 | (0.89-1.31) |  | 1.15 | (0.79-1.66) |

*CI* confidence interval, *EC* elemental carbon, *IQR* interquartile range, *OC* organic carbon, *OR* odds ratio.

^a^Adjusted for maternal age, birth year, season of conception, smoking, alcohol drinking, prepregnancy body mass index, current history of diabetes/gestational diabetes, infertility treatment, and parity.


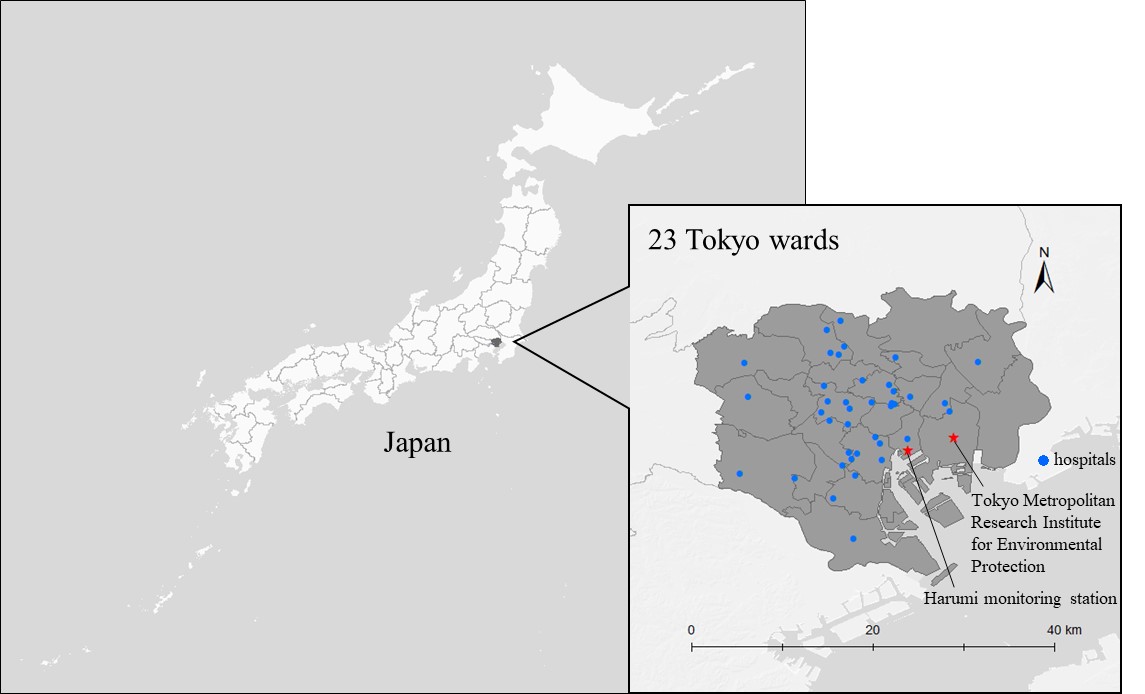


**Fig. S1.** Locations of monitoring stations and hospitals.
